# Knowledge mapping of global trends in DNA damage repair-related breast cancer research: a bibliometric study

**DOI:** 10.3389/fonc.2025.1562539

**Published:** 2025-05-29

**Authors:** Yajing Huang, Shumei Wei, Kaimin Hu, Xueping Xiang

**Affiliations:** ^1^ Department of Pathology, the Second Affiliated Hospital, Zhejiang University, School of Medicine, Hangzhou, China; ^2^ Department of Breast Surgery and Oncology, the Second Affiliated Hospital, Zhejiang University, School of Medicine, Hangzhou, China

**Keywords:** DNA damage repair, breast cancer, global trends, bibliometric study, CiteSpace, VOSviewer

## Abstract

**Background:**

Breast cancer remains one of the leading causes of cancer-related morbidity and mortality among women worldwide. Recent advancements in our understanding of DNA damage repair (DDR) mechanisms have shed light on their specific role in the pathogenesis, progression, treatment resistance, and prognosis of breast cancer. In this study, we conducted a bibliometric study to map the global trends in DDR-related breast cancer research.

**Methodology:**

A search of publications on DDR in breast cancer from 1990 to 2024 was conducted using the Web of Science Core Collection. A comprehensive bibliometric analysis of the data was performed using CiteSpace, VOSviewer, and Bibliometrix. Additionally, for clinical trial data, the databases ClinicalTrials.gov (https://www.clinicaltrials.gov) and the WHO International Clinical Trials Registry Platform (ICTRP) (https://trialsearch.who.int) were queried.

**Results:**

The results revealed a continuous and steady growth in the number of articles published in this area over the past three decades and showed that the USA had produced the highest number of publications in this field, while Harvard University had published the largest number of articles. Jonkers, Jos was found to be the most published author, with 39 documents. Analysis of the journals showed that *Cancer Research* ranked as the most published journal, while *Nature* was the most cited. Combined with the keyword co-occurrence analysis and co-citation analysis, it emerged that “Targeting the DNA repair defect as a therapeutic strategy”, published in *Nature* (IF = 64.8) in 2005, had accumulated 529 local citations, indicating that research topics have focused on treatment regimens. For clinical trials, 124 studies were initially sourced—108 from ClinicalTrials.gov and 16 from ICTRP. After repetitive and correlation-based screening, 43 trials specifically addressing 13 different DDR-related drugs in breast cancer were included.

**Conclusion:**

Overall, this study provides valuable insights into the current research achievements, latest advancements, and emerging global trends in DNA damage repair-related breast cancer research. With sustained clinical focus, more high-quality investigations combining DDR inhibitors with other treatment modalities are needed.

## 1 Introduction

Breast cancer is known to be the most common cancer and the leading cause of cancer-related deaths among women worldwide. The tumor is characterized by different molecular subtypes and heterogeneous biology, with a complex genomic landscape, and is also linked to impaired DNA damage repair (DDR) mechanisms (1–6), thus leading to varied therapeutic responses. DDR functions as a complex and coordinated mechanism that enables tissues and cells to cope with endogenous and exogenous DNA damage, thereby preventing genomic instability (7). Moreover, DDR pathways are essential for maintaining genomic stability, and their dysfunction can lead to tumorigenesis. The exploration of these pathways in the context of breast cancer research has gained momentum, as understanding these mechanisms can lead to the development of targeted therapies that exploit vulnerabilities in cancer research (3, 5).

When we look at DDR pathways and their association with breast cancer, we know that, as a malignant tumor, breast cancer harbors more DNA damage and replication stress, and the continual accumulation of DNA damages induces apoptosis and cell death. Different forms of DNA damage evoke different repair mechanisms and signaling pathways. There are six major repair pathways: nucleotide excision repair (NER) pathway, which deals with modified nucleotides; the base excision repair (BER) pathway, responsible for DNA single‐strand breaks; the mismatch repair (MMR) pathway, which addresses replication errors; the homologous recombination repair (HRR) pathway; the Fanconi anemia (FA) pathway, which repairs DNA interstrand crosslinks; the and nonhomologous end‐joining (NHEJ) pathway, which deals with DNA double‐strand breaks. Cell‐cycle checkpoints are also important for DDR to prevent DNA damage (7, 8). Previous studies have demonstrated that DDR alterations are common and crucial in various cancers. Germline mutations in DDR genes may predispose individuals to breast, ovarian, prostate, and pancreatic cancers (9–11). Mutations in Breast Cancer1/2 (BRCA1/2) are known to be important mechanisms responsible for HRR and are strongly associated with breast cancer risk. Breast BRCA1/2 also acts as predictive biomarkers for DNA-targeting therapies, such as poly(ADP‐ribosome) polymerase (PARP) inhibitors (12). In addition to germline BRCA1/2 mutations, other genes of vital importance that are associated with breast cancer, such as Ataxia Telangiectasia Mutated (ATM), Partner and Localizer of BRCA2 (PALB2), and Checkpoint Kinase 2 (CHEK2), also belong to the DDR pathway and are involved in the response to DDR‐targeting therapies (13). The complete DDR mutation status and other DDR gene mutations have not been well explored, especially in the somatic context. For anticancer therapies, downregulated expression of HRR proteins induces homologous recombination deficiency and increases the sensitivity to PARP inhibitors (14). However, when it comes to some other genes associated with different DDR pathways, it has been noted that loss of expression of MMR and NHEJ proteins, together with overexpression of NER pathway proteins, causes PARP inhibitor resistance (15–17). It has also been reported that DDR mutations are associated with an increased tumor mutation burden (TMB) in solid tumors (18). As far as we are concerned, intensive study is needed to manipulate the optimal expression of these DDR proteins. In the area of immunotherapy, DDR mechanisms and the role they play have been studied (19–21). Since breast cancer has been deemed an immunologically cold lesion, determining how to select potentially eligible patients and conduct therapeutic measures, including immunotherapy, is of great importance and requires further exploration (22). In summary, it is necessary to profoundly understand DDR alterations in breast cancer.

In terms of DDR’s specific clinical significance in breast cancer treatment, recent studies have shown that the benefit of PARP inhibitors extends beyond patients with germline BRCA1/2-associated metastatic breast cancer to those with somatic BRCA1/2 variants and to those with germline PALB2 alterations (23). In the phase III OlympiAD trial, olaparib improved progression-free survival (PFS) (hazard ratio [HR], 0.58 [95% CI, 0.43–0.80]; *p* <.001), doubled the objective response rate (ORR) (59.9% vs. 28.8%), and had a more favorable safety profile compared with physician’s choice treatment in patients with gBRCA1/2 variants who were pretreated with up to two lines of chemotherapy for metastatic breast cancer (24).

The bibliometric analysis serves as a valuable method used to shed light on the developmental status and research frontiers in specific areas and vast scenarios (25). It is recognized as an interdisciplinary science based on statistical and visualization techniques. A bibliometric analysis of research on multiple criteria decision-making depicted its developmental status and revealed its research focus in different periods (26, 27). In the biomedical area, bibliometric studies have also been widely conducted (28). Conducting a bibliometric study can provide a deep understanding of the landscape of DNA damage repair-related breast cancer, thereby inspiring interdisciplinary collaborations and identifying gaps in knowledge and potential areas for further investigation. In this study, we take a quantitative approach to analyze publication trends, influential works, and the evolution of research themes by employing bibliometric analysis to review literature related to DDR and breast cancer. This approach allows us to summarize complex information, highlight collaboration patterns, and identify emerging research frontiers.

## 2 Methodology

### 2.1 Search strategies and data collection

**Figure 1:**
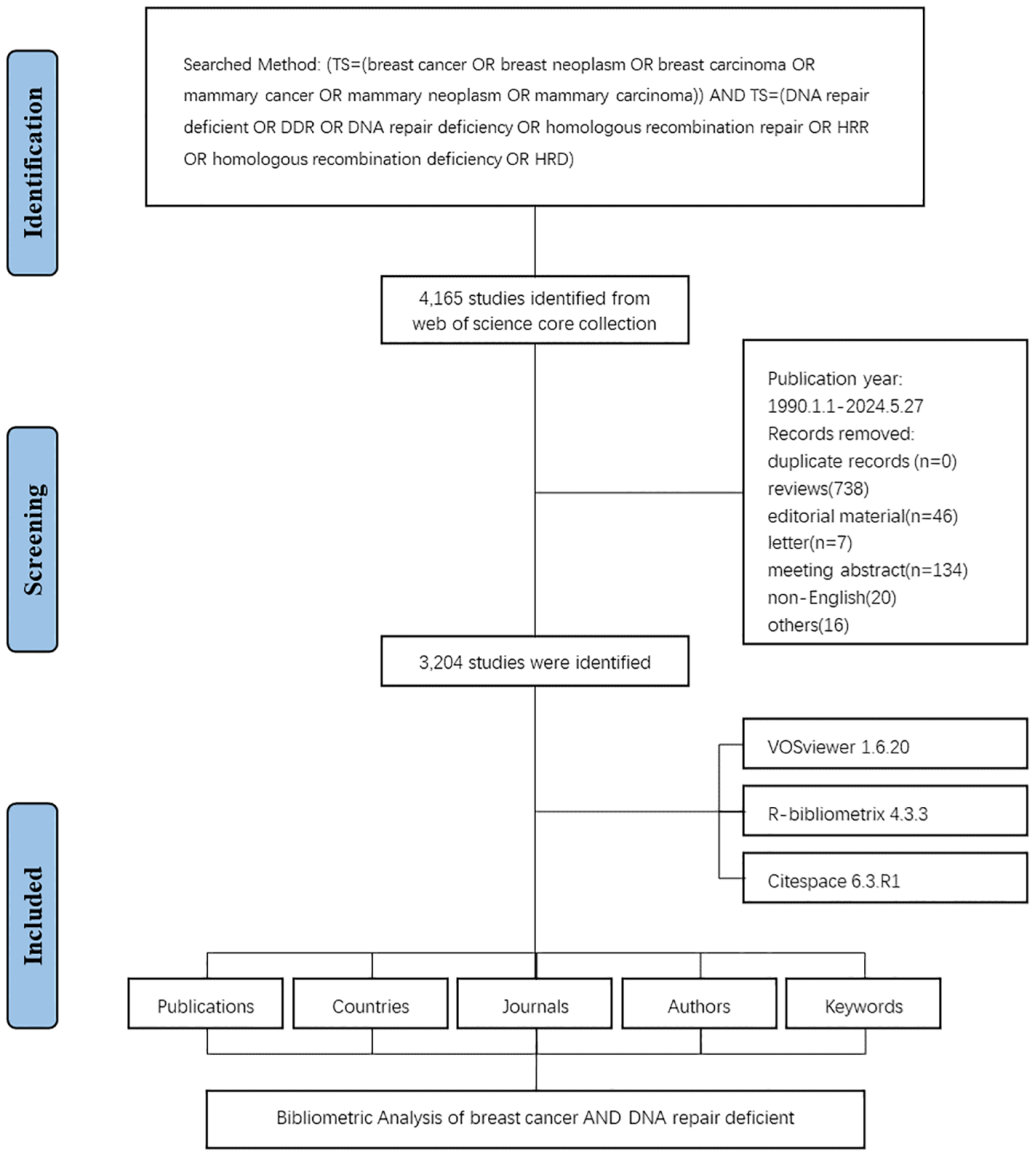
Flowchart of the literature screening process.

A comprehensive search was conducted on the Web of Science Core Collection (WoSCC). The search strategy included central terms such as “breast cancer” and “DNA damage repair” followed by detailed topic words: “breast cancer” or “breast neoplasm” or “breast carcinoma” or “mammary cancer” or “mammary neoplasm” or “mammary carcinoma” and “DNA damage repair” or “DDR” or “DNA repair deficiency” or “homologous recombination repair” or “HRR” or “homologous recombination deficiency” or “HRD”, covering publications from 1990 to 2024, which encompass key developments in the field. The publication language for this study was set to English. Among various types of documents, only articles were considered. To avoid discrepancies from database updates, the literature retrieval was performed on a single day (22 May 2024). All data were collected in the text format. The collected information included the number of publications and citations, titles, author details, institutions, countries/regions, keywords, and journals, all of which were used for further bibliometric analysis. A total of 3,204 eligible publications were included in the present study. The flowchart for data screening is shown in **Figure 1**.

### 2.2 Bibliometric analysis

We exported relevant data from the retrieved literature titles and used Microsoft Excel to identify and calculate bibliometric indicators. These indicators encompassed key aspects of the publications, including annual publication quantity, citation frequency, average citation frequency, journal name, journal impact factor, publishing country/region, publishing institution, and corresponding author. By leveraging Excel, researchers were able to efficiently organize and analyze the bibliometric data. During the visualization analysis process, we utilized three powerful bibliometric tools to conduct a comprehensive analysis of the academic data. These tools were VOSviewer (version 1.6.20), CiteSpace (version 6.3. R1), and an online bibliometric tool (https://bibliometric.com/). VOSviewer is a multifunctional software tool that plays a key role in mapping institutional collaborations, author collaborations, co-authorship, citation, and co-citation (doi: 10.1007/s 11192-009-0146-3) (29). Using VOSviewer, we were able to visualize and explore complex networks of collaboration and relationships within the academic field, gaining deeper insights into the interconnections between authors, institutions, and publications. To gain a deeper understanding of emerging trends and research hotspots in our field, we used VOSviewer for keyword co-occurrence analysis and CiteSpace for keyword burst detection (30).

Using CiteSpace 6.1.R3, we conducted a keyword co-occurrence analysis with time slicing as the parameter, covering the period from January 1996 to May 2024 (the first research in this field was published in 1996). The time slice was set to 1 year. The node type was set to keywords, with the threshold for each segment being the top 5 names. Pruning was set to pathfinder and pruning merged the network. Based on the parameter settings, a visual analysis was performed to generate a keyword timeline in the research field of “DNA repair defects in breast cancer”.

The collected data were analyzed using the bibliometric software of VOSviewer and Bibliometrix to generate visual maps illustrating publication trends, thematic clustering, and citation networks. The size of the nodes represents the number of publications, the thickness of the lines indicates the strength of the link, and the color of the nodes corresponds to different clusters or time periods. Key metrics, such as the number of publications, citations, and the H-index of authors and institutions, were considered to assess research impact and predict future achievements. In our study, the H-index of individuals and journals was obtained from WoSCC.

### 2.3 Clinical trial analysis

Data were extracted from ClinicalTrials.gov and the World Health Organization’s International Clinical Trials Registry Platform (ICTRP). A search was conducted using the parameters “DDR OR DNA damage repair” and “breast cancer” on 11 November 2024. Initially, 108 trials from ClinicalTrials.gov and 16 from ICTRP were retrieved. The author then performed a repetitive and correlation-based screening, excluding trials not related to breast cancer or DDR-related drugs. Afterward, data from the two databases were amalgamated, and duplicate trials were removed. Finally, 43 trials investigating DDR-related drugs for breast cancer were included in our analysis. Information on relevant variables, such as study design, status, phase, conditions, interventions, outcomes, and commencement dates, was reviewed.

## 3 Results

### 3.1 The overall situation of the research field

**Figure 2:**
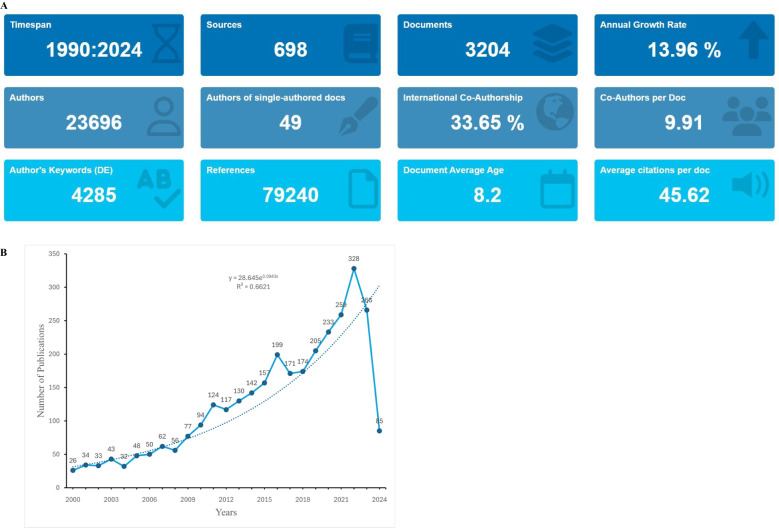
**(A)** Overview of publications on DNA repair deficiency in breast cancer. **(B)** Annual number of publications.

Our study examined 3,204 publications, all of which are articles. The analysis showed that 23,696 authors from 14,417 institutions across 601 countries/regions contributed to the production of these manuscripts. These works were published in 698 journals and cited 79,240 references (**Figure 2A**).

### 3.2 Publication trends

The analysis revealed a steady increase in the number of publications focusing on DNA damage repair in breast cancer over the study period from January 1996 to May 2024. Publications from 2000 to 2024 were analyzed based on the search strategy. **Figure 2B** illustrates the overall trend in the number of publications from 2000 to 2024, with a notable spike in research output after 2014. This trend aligns with the growing focus on personalized medicine and targeted therapy in oncology.

**Figure 3:**
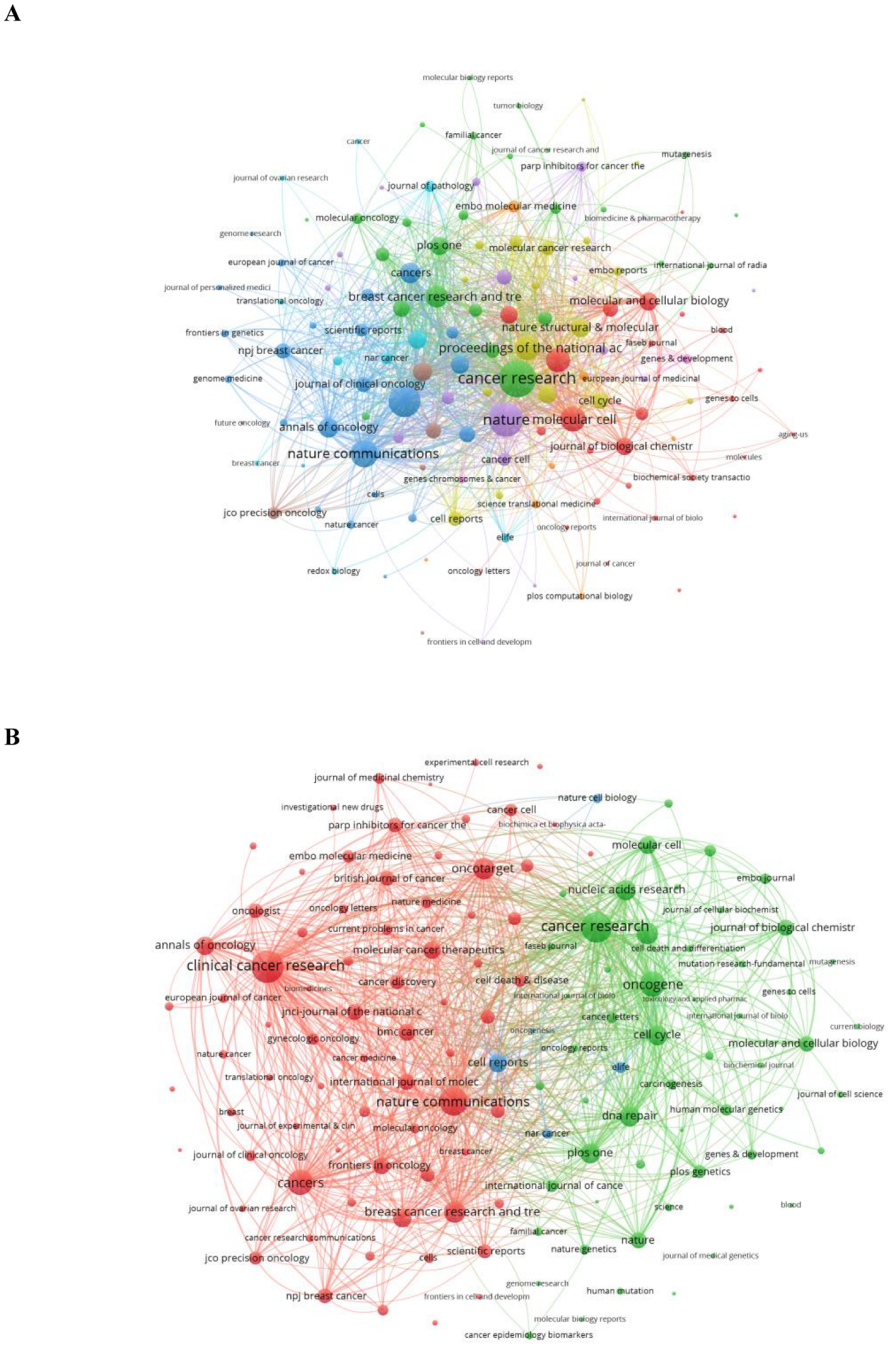
**(A)** Co-occurrence network of journals. **(B)** Coupling network of journals.

The high-impact journals in this research field are listed in **Table 1**. Among the publications, the most cited article was titled “Targeting the DNA repair defect in BRCA mutant cells as a therapeutic strategy” and was published in *Nature* (impact factor (IF) = 64.8) in 2005, which accumulated a total of 4,753 citations (doi: 10.1038/nature03445). The article with the highest IF (158.5) was titled “Niraparib maintenance therapy in platinum-sensitive, recurrent ovarian cancer”, published in the *New England Journal of Medicine* (IF = 158.5) in 2016, which accumulated a total of 1,670 citations (doi: 10.1056/NEJMoa1611310). We also analyzed the distribution and co-occurrence of journals containing this topic. The co-occurrence networks of journals include 135 journals with at least five occurrences. The three key journals with the highest total link strength in co-occurrence networks were *Cancer Research* (1,349), *Nature* (1,137), and *Clinical Cancer Research* (989) (**Figure 3A**). Furthermore, the coupling networks of journals also consist of 135 journals with at least five couplings. The three key journals with the highest total link strength in the co-occurrence networks were *Clinical Cancer Research* (90,966), *Cancer Research* (82,308), and *Nature Communications* (71,474) (**Figure 3B**).

**Table 1:** Bibliometric indicators of high-impact journals.

Journal	H_index	IF	JCR_Quartile	PY_start	TP	TP_rank	TC	TC_rank
Cancer Research	56	11.2	Q1	1998	119	1	6,997	2
Proceedings of the National Academy of Sciences of the United States of America	40	11.1	Q1	1993	57	8	4,951	3
Clinical Cancer Research	39	11.5	Q1	1995	86	2	3,720	8
Oncogene	35	8.0	Q1	1995	75	3	3,494	9
Nature Communications	34	16.6	Q1	2011	63	7	1,858	17
Journal of Biological Chemistry	31	4.8	Q2	1996	40	14	3,893	5
Breast Cancer Research and Treatment	24	3.8	Q2	2002	65	5	1,707	18
Molecular Cancer Therapeutics	24	5.7	Q2	2005	40	15	942	38
Molecular Cell	23	16.0	Q1	1998	29	21	3,886	6
Nature	23	64.8	Q1	1997	25	26	7,544	1
PLOS One	23	3.7	Q3	2009	64	6	1,551	21
Cell Cycle	22	4.3	Q2	2004	46	11	976	34
Nucleic Acids Research	21	14.9	Q1	1999	38	16	3,005	12
Oncotarget	21	NA	NA	2013	54	9	1,112	32
International Journal of Cancer	20	6.4	Q1	1995	30	20	1,264	24
Annals of Oncology	19	50.5	Q1	2011	28	22	1,569	20
Breast Cancer Research	19	7.4	Q1	2006	34	19	1,133	29
Cell Reports	19	8.8	Q1	2014	26	24	841	39
BMC Cancer	18	3.8	Q2	2004	37	17	644	50
Molecular and Cellular Biology	18	5.3	Q2	1999	22	32	2,402	14

*H_index*, the h-index of the journal (which measures both the productivity and citation impact of the publications); *IF*, impact factor (indicating the average number of citations to recent articles published in the journal). *JCR_Quartile*, the quartile ranking of the journal in the Journal Citation Reports (indicating the journal’s ranking relative to others in the same field [Q1: top 25%, Q2: 25%–50%, Q3: 50%–75%, Q4: bottom 25%]); *TP*, total publications; *TP_rank*, rank of total publications; *TC*, total citations; *TC_rank*, rank of total citations; *Average Citations*, the average number of citations per publication; *PY_start*, publication year start (indicating the year the journal started publication).

### 3.3 Analysis of the countries

**Figure 4:**
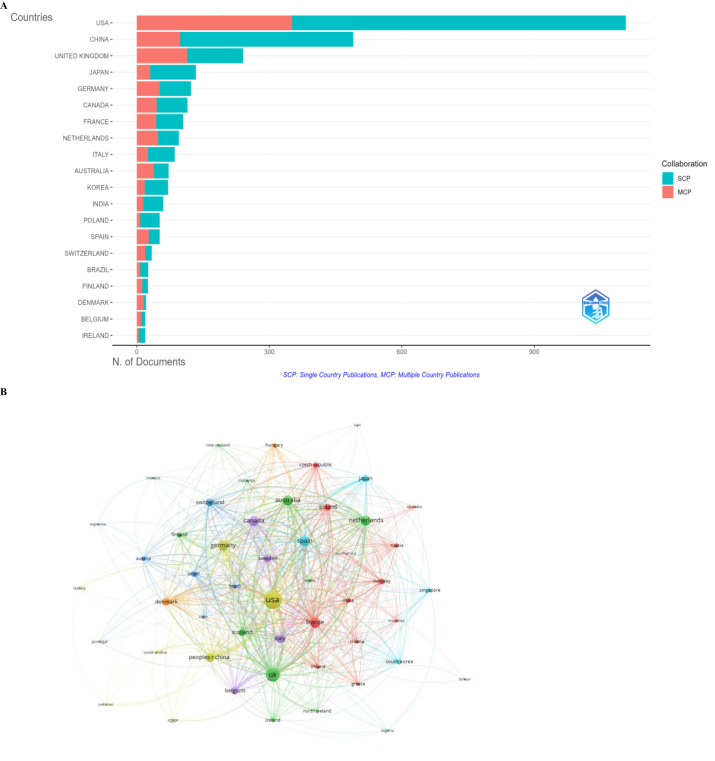
**(A)** Distribution of corresponding author’s publications by country (R Bibliometrix). **(B)** Visualization map showing collaboration among different countries.

In total, articles published in this field were contributed by 50 countries/regions. The top 10 most productive countries generated 2,568 articles, accounting for 80% of all papers worldwide. The USA was the most productive country, with the highest number of articles (*n* = 1,107), followed by China (*n* = 490) and the UK (*n* = 241). Among the top 10 productive countries/regions, as shown in **Figure 4A**; **Table 2**, the USA held the leading position in terms of average citations (*n* = 57.7), reflecting the significant impact and widespread interest generated by its research contributions. Among the 38 countries involved in international collaborations with at least one article, the USA had the highest number of collaborations (1,298), followed by the UK (813) and France (468) (**Table 3**). The data were analyzed using VOSviewer (**Figure 4**).

**Table 2:** Publication and citation profiles of leading countries.

Country	Articles	Freq	MCP ratio	TP	TP rank	TC	TC rank	Average citations
USA	1,107	0.346	0.317	5,379	1	63,912	1	57.7
China	490	0.153	0.200	2,019	2	7,796	4	15.9
UK	241	0.075	0.473	1,182	3	27,806	2	115.4
Japan	134	0.042	0.224	771	6	2,540	10	19
Germany	123	0.038	0.423	793	5	4,887	5	39.7
Canada	115	0.036	0.391	659	8	3,925	6	34.1
France	105	0.033	0.419	821	4	3,767	7	35.9
Netherlands	95	0.03	0.505	622	9	7,828	3	82.4
Italy	86	0.027	0.291	568	10	2,603	9	30.3
Australia	72	0.022	0.542	686	7	2,524	11	35.1
Korea	71	0.022	0.254	363	12	1,567	13	22.1
India	60	0.019	0.250	175	16	704	19	11.7
Poland	52	0.016	0.154	235	13	857	15	16.5
Spain	52	0.016	0.519	488	11	1,758	12	33.8
Switzerland	34	0.011	0.559	187	15	1,411	14	41.5
Brazil	26	0.008	0.308	121	20	393	22	15.1
Finland	25	0.008	0.480	190	14	825	17	33
Denmark	21	0.007	0.762	125	18	2,638	8	125.6
Belgium	19	0.006	0.526	153	17	531	21	27.9
Ireland	19	0.006	0.211	73	24	847	16	44.6

*Articles*, publications of corresponding authors only; *Freq*, frequency of total publications; *MCP ratio*, proportion of multiple country publications; *TP*, total publications; *TP rank*, rank of total publications; *TC*, total citations; *TC rank*, rank of total citations; *Average citations*, the average number of citations per publication.

**Table 3:** Total link strength in collaboration networks.

Id	Country	Documents	Citations	Total link strength
87	USA	1,442	90,259	1,298
84	UK	414	44,934	813
24	France	189	12,795	468
72	Spain	137	9,219	434
26	Germany	226	13,683	430
10	Canada	213	13,214	396
48	Netherlands	179	17,092	396
2	Australia	133	10,820	386
37	Italy	162	9,753	345
57	China	602	13,692	328
75	Sweden	64	8,768	262
18	Denmark	66	7,451	252
76	Switzerland	88	6,013	243
6	Belgium	55	6,857	231
65	Scotland	48	6,984	218
38	Japan	211	7,602	197
59	Poland	85	6,919	177
36	Israel	49	4,845	145
53	Norway	29	4,968	144
17	Czech Republic	34	2,625	124

### 3.4 Authors’ impact and co-occurrence network

As shown in **Table 4**, among the authors with high publication and citation impact, Jonkers, Jos ranked first, with the highest h-index (31), which reflects both productivity and citation impact, and the highest g-index (49), which gives more weight to highly cited articles. He was followed by Ashworth, Alan, who held the second-highest h-index (22), and Rottenberg, Sven, who had the second-highest g-index (31). In terms of total publications (TP) and total citations (TC), Jonkers, Jos also ranked first.

**Table 4:** Publication and citation profiles of high-impact authors.

Authors	H_index	g-index	m-index	PY start	TP	TP Frac	TP rank	TC	TC rank
Jonkers, Jos	31	49	1.82	2008	49	5.43	1	7,192	1
Ashworth, Alan	22	27	1.16	2006	27	3.21	3	5,006	3
Lord, Christopher J.	21	27	1.11	2006	27	2.80	4	4,017	6
O’Connor, Mark J.	21	27	1.11	2006	27	1.72	5	4,748	4
Rottenberg, Sven	21	29	1.24	2008	29	2.78	2	3,589	7
Swisher, Elizabeth M.	18	26	1.50	2013	26	1.55	6	3,555	9
D’Andrea, Alan D.	17	20	0.94	2007	20	2.81	9	3,194	10
Ellis, Ian O.	16	20	1.23	2012	20	1.77	10	673	42
Reis-Filho, Jorge S.	16	25	1.07	2010	25	1.68	7	1,240	30
Xia, Bing	16	20	0.84	2006	20	1.70	13	2,302	14
Bouwman, Peter	15	18	0.88	2008	18	2.12	17	2,241	16
Jasin, Maria	15	20	0.79	2006	20	2.83	11	3,581	8
Garber, Judy E.	14	16	0.88	2009	16	1.09	23	4,125	5
Madhusudan, Srinivasan	14	21	1.17	2013	21	1.93	8	609	44
Powell, Simon N.	14	18	0.78	2007	18	2.86	19	1,279	28
Rakha, Emad A.	14	17	1.17	2013	17	1.60	20	526	47
Green, Andrew R.	13	16	1.00	2012	16	1.50	24	463	48
Konstantinopoulos, Panagiotis A.	13	14	0.87	2010	14	1.10	29	1901	20
Nik-Zainal, Serena	13	15	1.30	2015	15	1.44	28	2903	11
Weigelt, Britta	13	19	0.93	2011	19	1.20	16	812	37

*H_index*, the h-index of the journal which measures both the productivity and citation impact of the publications); *g_index*, The g-index of the journal (which gives more weight to highly cited articles); *m_index*, The m-index of the journal (which is the h-index divided by the number of years since the first published paper); *TP*, total publications; *TP rank*, rank of total publications; *TC*, total citations; *TC rank*, rank of total citations; *Average citations*, the average number of citations per publication; *PY start*; publication year start, indicating the year the journal started publication.

**Figure 5:**
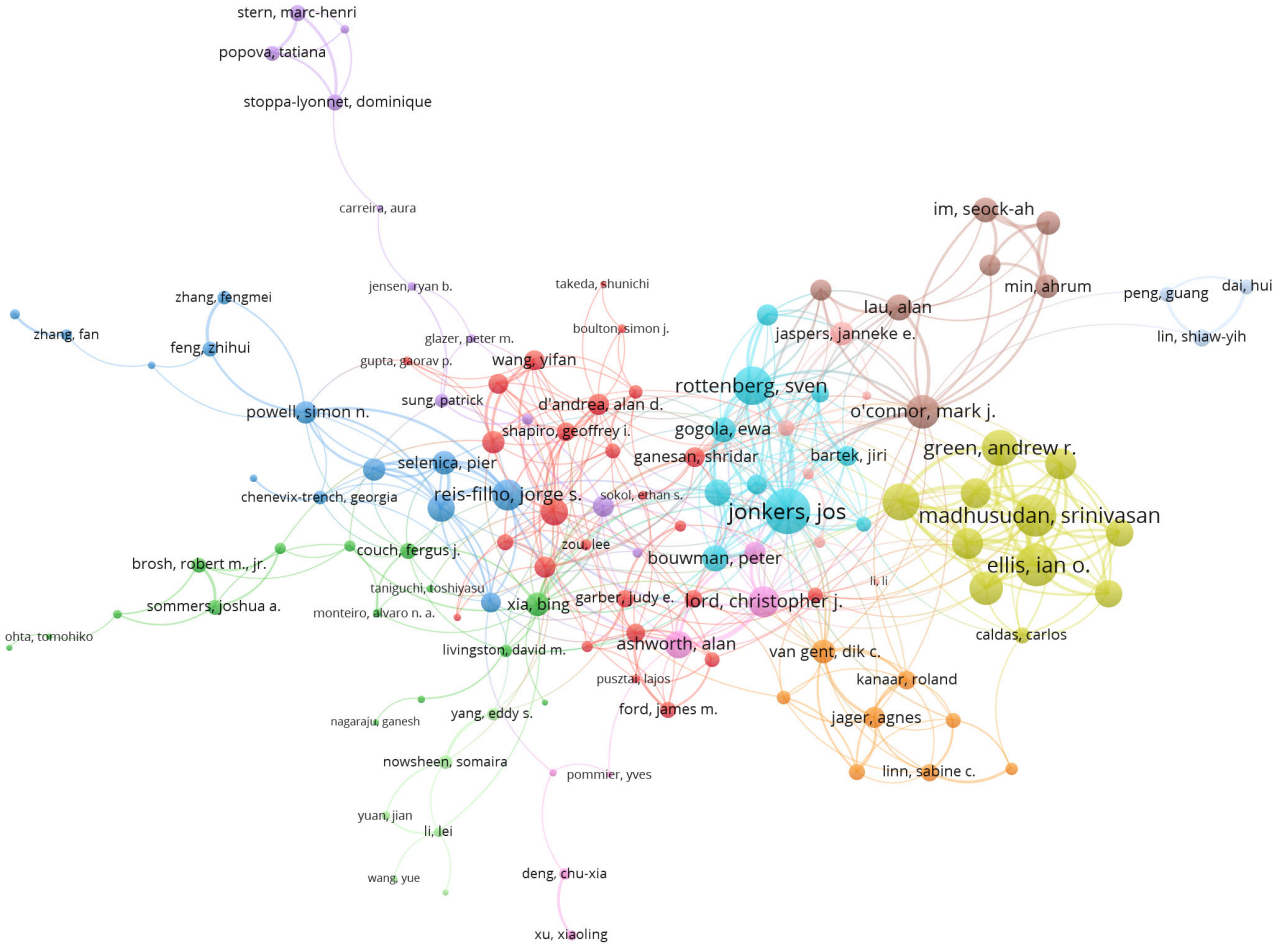
Visualization map showing collaboration among different authors.

For the co-occurrence network of leading authors and institutions, we observed that a relatively small number of authors contributed significantly to the body of literature. The study identified key institutions that pioneered DDR research, emphasizing their role in advancing the field through influential publications and interinstitutional collaborations. Among the 290 authors engaged in international collaborations with at least five articles, Jonkers, Jos had the highest number of collaborations with other countries (114), followed by Ellis, Ian O. (95) and Madhusudan, Srinivasan (95). A visualization map illustrating collaboration among different authors revealed that Jonkers, Jos ranked first in total link strength (114), based on 39 documents and 4,229 citations (**Figure 5**; **Table 5**).

**Table 5:** Collaboration network among different authors.

ID	Author	Documents	Citations	Total link strength
7947	Jonkers, Jos	39	4,229	114
4499	Ellis, Ian O.	19	630	95
10903	Madhusudan, Srinivasan	21	609	95
14866	Rottenberg, Sven	25	2,493	81
14280	Rakha, Emad A.	17	526	75
5867	Green, Andrew R.	15	420	70
17014	Takabe, Kazuaki	20	324	65
13049	Oshi, Masanori	18	275	64
12820	O’Connor, Mark J.	22	4;306	62
23	Abdel-Fatah, Tarek M. A.	9	286	61
19676	Yan, Li	14	273	56
594	Arora, Arvind	8	211	55
14473	Reis-Filho, Jorge S.	20	1,022	55
10575	Lord, Christopher J.	22	3,486	53
2414	Chan, Stephen Y. T.	8	197	52
12140	Moseley, Paul M.	7	191	50
4526	Endo, Itaru	13	168	49
124	Agarwal, Devika	7	208	43
17487	Tokumaru, Yoshihisa	9	133	43
18852	Weigelt, Britta	15	599	42

### 3.5 The article number from different institutions and co-occurrence networks

**Figure 6:**
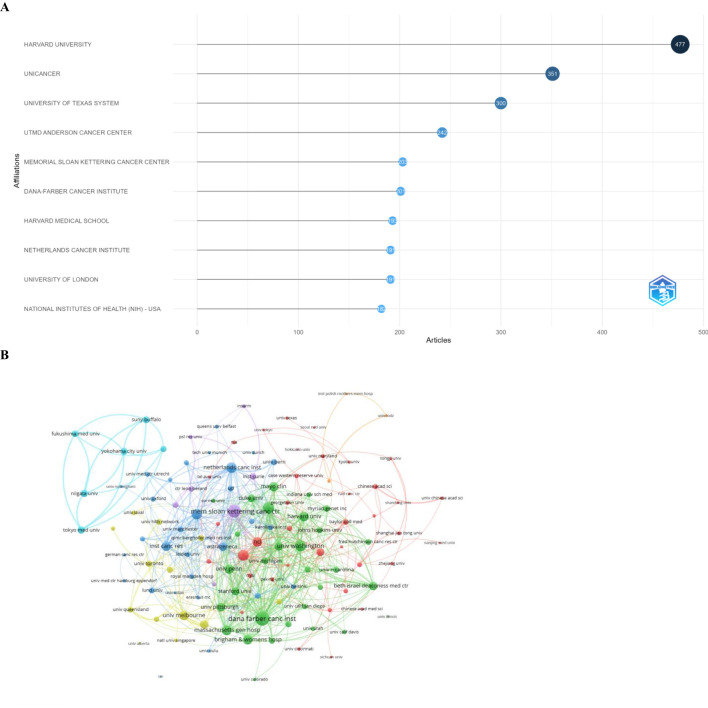
**(A)** Top 10 institutions by article number and rank (R Bibliometrix). **(B)** Visualization map showing collaboration among different institutions.

Among the top 10 institutions ranked by article count in this field, Harvard University ranked first with 477 articles, followed by UNICANCER, France with 351 articles and the University of Texas System with 300 articles (**Figure 6A**). Of the 75 institutions engaged in international collaborations (each with a minimum of two articles), the Dana-Farber Cancer Institute (Dana Farber Canc Inst) had the highest number of collaborations (298), followed by Memorial Sloan Kettering Cancer Center (Mem Sloan Kettering Canc Ctr) (241) and Harvard Medical School (Harvard Med Sch) (186) (**Figure 6B**; **Table 6**).

**Table 6:** Collaboration among different institutions.

ID	Organization	Documents	Citations	Total link strength
698	Dana Farber Canc Inst	80	10,366	298
1944	Mem Sloan Kettering Canc Ctr	113	14,353	241
1128	Harvard Med Sch	70	4,068	186
3578	Univ Washington	47	5,222	184
3528	Univ Texas Md Anderson Canc Ctr	96	9,600	173
2148	Netherlands Canc Inst	93	9,757	165
3093	Univ Cambridge	69	13,923	164
1877	Massachusetts Gen Hosp	43	5,934	146
278	Brigham and Women’s Hosp	41	4,433	139
2143	NCI	92	4,911	139
1135	Harvard Univ	74	10,018	134
3353	Univ Melbourne	44	1,902	131
3437	Univ Penn	40	3,986	128
1419	Inst Canc Res	76	14,077	122
1896	Mayo Clin	49	3,521	122
238	Beth Israel Deaconess Med Ctr	23	2,500	110
3441	Univ Pittsburgh	49	2,482	110
2361	Peter Maccallum Canc Ctr	31	3,224	105
139	AstraZeneca	45	4,948	102
3091	Univ Calif San Francisco	29	3,265	102

### 3.6 The analysis of keywords

**Figure 7:**
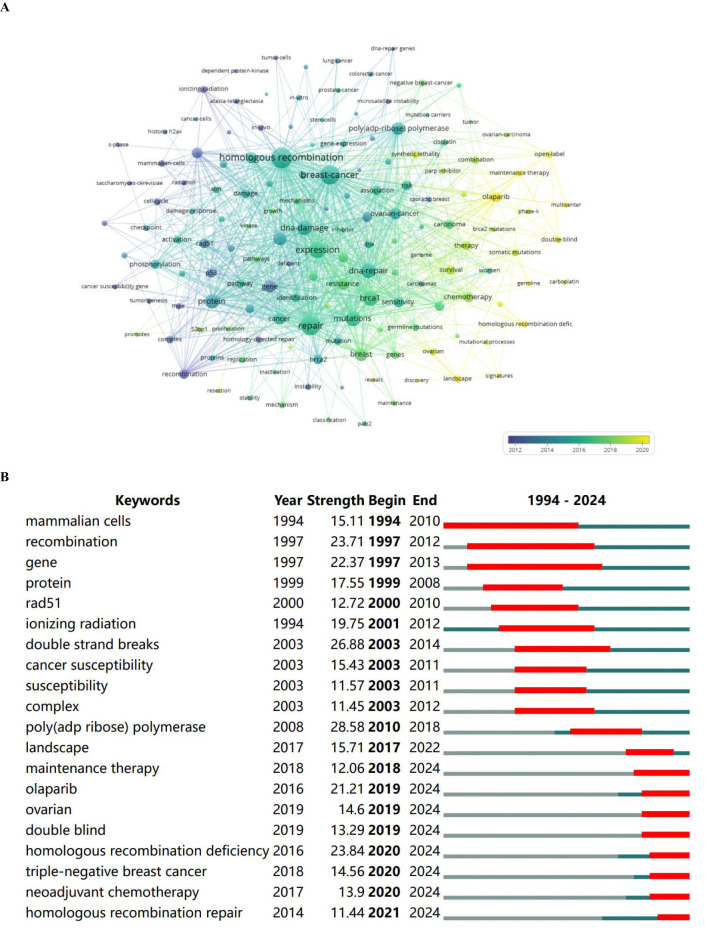
**(A)** Visual representation of the keyword co-occurrence network. **(B)** Top 20 keywords with the strongest citation bursts (CiteSpace).

A comprehensive keyword analysis of the selected articles was performed using”Author Keywords” from the Biblioshiny application and “Keywords Plus” provided by the VOSviewer application. In total, 175 keywords with a minimum of 25 occurrences were identified. Upon comparing the results from these two sources, “Keywords Plus” was found to provide more accurate results and was thus used as the primary data source for the analysis. Among the top 20 keywords with the highest total link strength and co-occurrence frequency, the top three were “homologous recombination”, “repair”, and “breast cancer” (**Figure 7A**; **Table 7**). Furthermore, among the top 20 keywords with the strongest citation bursts, the most significant burst belongs to “poly(ADP-ribose) polymerase”. Notably, since 2017, the keywords “landscape”, “maintenance therapy”, “olaparib”, “ovarian” “double-blind”, “homologous recombination deficiency”, “triple-negative breast cancer”, “neoadjuvant chemotherapy”, and “homologous recombination repair” have appeared more prominently, indicating promising developments (**Figure 7B**).

**Table 7:** Data of keyword co-occurrence network.

ID	Keyword	Occurrences	Total link strength
2323	Homologous recombination	661	3,248
4300	Repair	600	2,954
614	Breast cancer	667	2,856
1754	Expression	411	1,996
3290	Mutations	352	1,758
550	BRCA1	346	1,746
1470	DNA repair	345	1,714
1447	DNA damage	310	1,602
4041	Protein	310	1,529
827	Cells	284	1,338
610	Breast	266	1,311
3843	Poly(ADP-ribose) polymerase	217	1,202
3552	Ovarian cancer	224	1,105
676	Cancer	244	1,089
4355	Resistance	197	1,060
1992	Gene	207	1,034
1451	DNA damage response	199	923
1515	Double-strand breaks	188	883
4510	Sensitivity	147	808
4794	Susceptibility	169	804

### 3.7 The analysis of references

**Figure 8:**
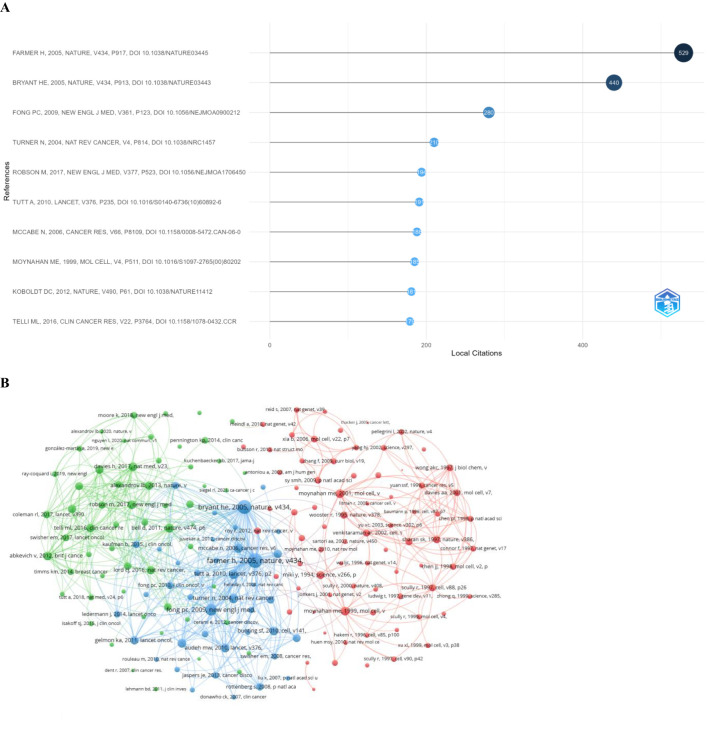
**(A)** The top 10 co-cited references in the field of DNA damage repair-related breast cancer. **(B)** Co-occurrence analysis of co-cited references cited more than 50 times.

Based on the co-occurrence analysis of references cited more than 50 times, we selected the top 10 co-cited references in the field of DNA damage repair-related breast cancer. Among these, the article titled “Targeting the DNA repair defect in BRCA mutant cells as a therapeutic strategy”—published in *Nature* (IF = 64.8) in 2005—received 529 local citations. (doi: 10.1038/nature03445) (**Figures 8A, B**).

### 3.8 Clinical trial analysis

**Figure 9:**
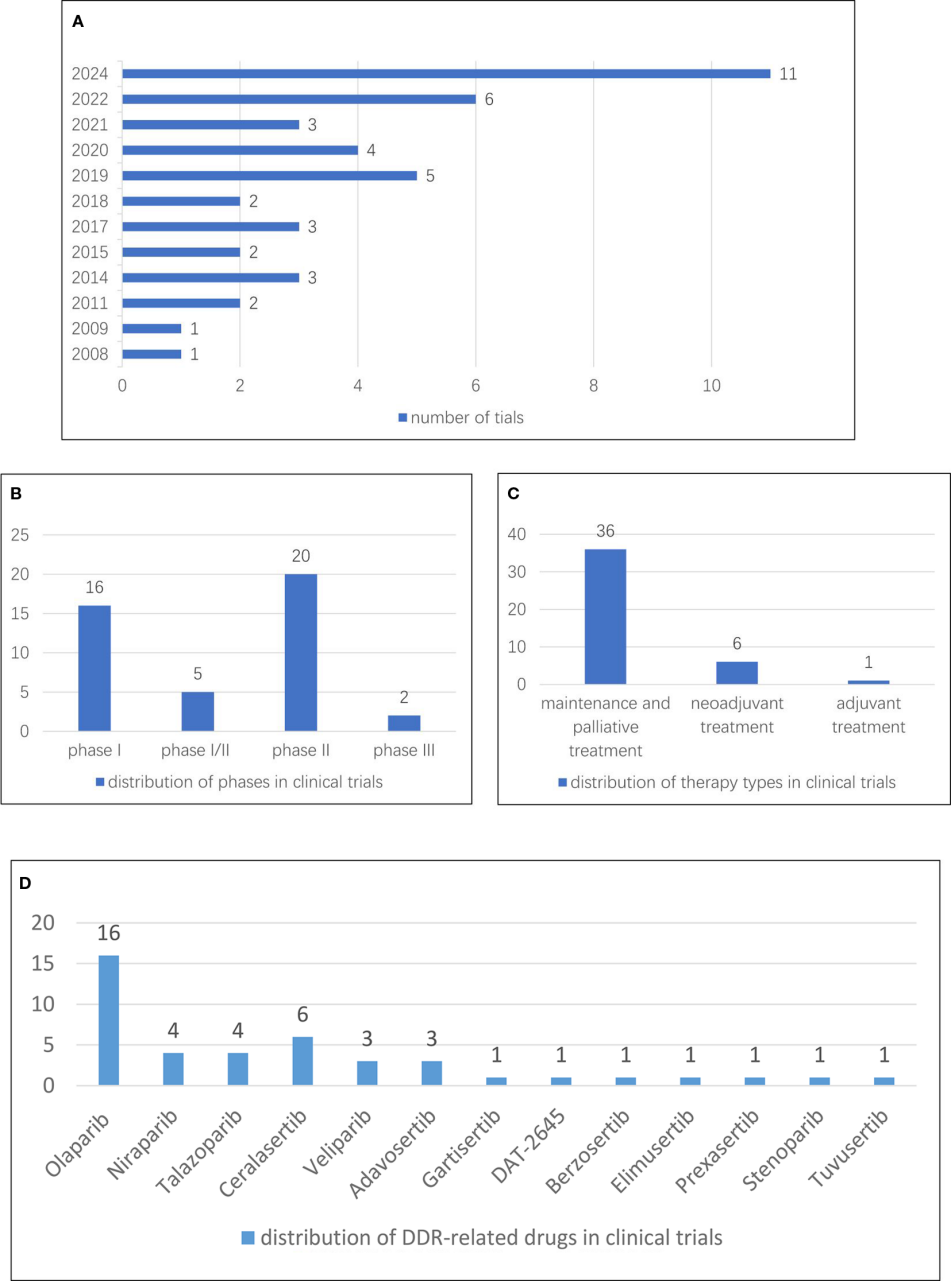
**(A)** Annual distribution of a number of clinical trials. **(B)** Distribution of phases in clinical trials. **(C)** Distribution of therapy types in clinical trials. **(D)** Distribution of DDR-related drugs in clinical trials.

The first clinical trial for DDR-related drugs for breast cancer was conducted in 2008 and was titled “AZD2281 Plus Carboplatin to Treat Breast and Ovarian Cancer”. Specifically, this phase I trial aimed to determine the safety and toxicity of the combination of AZD2281 (KU-0059436) and carboplatin in patients with recurrent BRCA1/2-associated or familial breast and ovarian cancer, recurrent low-genetic-risk serous ovarian cancer, and recurrent low-genetic-risk triple-negative breast cancer. The trial also aimed to assess biochemical changes in PARP and H2A histone family member X (H2AX) activity in mononuclear cells, as well as tumor response to treatment. The corpus of clinical trials for DDR-related drugs in breast cancer has expanded since that time. A notable peak occurred in 2024, with 11 trials conducted (**Figure 9A**). Among the 43 enumerated clinical trials, phase II trials represented the largest category, totaling 20, followed by 16 phase I trials (**Figure 9B**). Regarding therapy types, maintenance and palliative treatment trials were the most common, with 36 trials, followed by neoadjuvant treatment trials, which accounted for six (**Figure 9C**). In terms of DDR-related drug distribution across the clinical trials, a total of 13 different drugs were studied, with olaparib being the most frequently used in 16 trials, followed by ceralasertib in six trials (**Figure 9D**).

Additionally, among these 43 clinical trials, we got the information that the National Cancer Institute (NCI) sponsored the most trials (11), followed by Sanofi with six. Regarding treatment strategies, efficacy, and outcomes, 11 trials with completed study status were identified. Among them, nine trials involved combination treatment strategies (combination of DDR-related drugs with chemotherapy or immunotherapy). Moreover, of these 11 completed trials, four were categorized as phase I, in which drug efficacy was measured by biochemical changes in tumor cells or participant response rates. The remaining six trials were phase II, and one was phase II; their outcomes were measured using pathological complete response rate (PCR) (in two neoadjuvant treatment trials), ORR, overall survival (OS), or PFS.

## 4 Discussion

### 4.1 General information related to publications

It is widely known that breast cancer is a multifaceted malignant disease influenced by genetic, environmental, and lifestyle factors. Recent studies have increasingly focused on the role of DDR mechanisms in the etiology, progression, and treatment responses of breast cancer. To shed light on the key findings and emerging themes in this area, we summarized various aspects identified through this bibliometric study. A search of publications on DDR for breast cancer from 1990 to 2024 was conducted, and the research revealed that the USA has produced the largest number of publications in this field, which might be explained by its scientific infrastructure and government funding, while Harvard University published the highest number of articles. Jonkers, Jos was found to be the most published author, with 39 documents. Analysis of the journals showed that *Cancer Research* ranked as the most published journal, while *Nature* was the most cited. Through keyword co-occurrence and co-citation analyses, it was found that the study titled “Targeting the DNA repair defect as a therapeutic strategy”, published in *Nature* (IF = 64.8) in 2005, received 529 local citations. This indicates that research has increasingly focused on treatment regimens. Overall, the study provides valuable insights into current research achievements, latest advancements, and emerging global trends in DNA damage repair-related breast cancer research.

For key findings, our analysis highlights major trends in DNA repair deficiency in breast cancer. A total of 175 keywords, each occurring at least 25 times, were identified (15, 31–48). The “Keywords Plus” was observed to provide more accurate results. Among the top 20 keywords with the highest total link strength and co-occurrence frequency, the three most prominent were “homologous recombination”, “repair”, and “breast cancer”. Furthermore, among the top 20 keywords with the strongest citation bursts, the most significant citation burst was observed for “poly(ADP-ribose) polymerase”. Recent studies show that since 2017, the keywords “landscape”, “maintenance therapy”, “olaparib”, “ovarian” “double-blind”, “homologous recombination deficiency”, “triple-negative breast cancer”, “neoadjuvant chemotherapy”, and “homologous recombination repair” have become more prominently featured.

### 4.2 Exploration and analysis of DDR mechanisms

According to the “Keywords Plus” analysis, the top three keywords with the highest total link strength and co-occurrence frequency were “homologous recombination”, “repair”, and “breast cancer”. “Homologous recombination” is the most well-known DDR pathway in breast cancer, particularly in relation to BRCA1 and BRCA2 mutations, which significantly increase the risk of the disease. Studies have shown that defects in HR can lead to unrepairable double-strand breaks, promoting tumor growth (38–42, 49). For the keywords “repair” and “breast cancer”, we reviewed the literature and synthesized information on DNA damage repair from previous studies to evaluate the role of DNA in breast cancer. DDR pathways are crucial for maintaining genomic stability, and their dysregulation can lead to tumorigenesis. For example, the BER pathway repairs small base lesions caused by oxidation or alkylation, which, if accumulated, may contribute to the initiation of breast cancer (50). NER is essential for removing bulky DNA adducts that can form in response to environmental carcinogens (51). Besides the BRCA genes, other genes involved in DDR pathways—such as PALB2, RAD51, and ATM—have also been implicated in hereditary breast and ovarian cancer syndromes. Furthermore, recent genome-wide association studies (GWAS) have identified single nucleotide polymorphisms (SNPs) associated with an increased risk of breast cancer (52), suggesting that variations in DDR capacity may contribute to breast cancer susceptibility.

Through literature screening during this bibliometric investigation, we uncovered underexplored areas within the domain of DNA damage repair mechanisms in breast cancer, which may guide future research directions in this field.

### 4.3 Clinical significance and clinical trials of breast cancer patients with DDR deficiency

Among the top 20 keywords with the strongest citation bursts, the most significant was observed for “poly(ADP-ribose) polymerase”, beginning in 2010. Importantly, the data indicate that since 2017, keywords such as “landscape”, “maintenance therapy”, “olaparib”, “ovarian” “double-blind”, “homologous recombination deficiency”, “triple-negative breast cancer”, “neoadjuvant chemotherapy”, and “homologous recombination repair” have gained increasing prominence. Notably, the keywords “maintenance therapy”, “olaparib”, and “neoadjuvant chemotherapy” pertain to treatment regimen regimens. Olaparib, a poly(ADP-ribose) polymerase inhibitor, has demonstrated significant efficacy in patients with hereditary breast cancer (38). Homologous recombination is a multifactorial process involving numerous proteins beyond BRCA1 and BRCA2. Genomic alterations in the genes encoding these proteins can impair HRR, making them potential targets for synthetic lethality using PARP inhibitors and other DDR-targeting agents (23). The keywords “ovarian” and “triple-negative breast cancer” prominently highlight the associated tumor types. Since 2019, the emergence of the keyword “double-blind” indicates an increasing number of clinical trials in this area. Overall, the top 20 keywords with the strongest citation bursts suggest that PARP inhibitors play a significant role in the treatment of breast cancer with DDR deficiencies, especially in triple-negative breast cancer. To develop individual treatment regimens, evidence-based medicine is essential; therefore, more high-quality clinical are urgently needed.

In the context of clinical trials, after examining databases and screening information on clinical studies involving DDR-related drugs for breast cancer, we found that 43 relevant trials have been registered since 2008. Notably, 2024 has seen a significant increase, with 11 trials registered so far. Among these, 36 trials (84% of the total) focused on advanced tumors and involved maintenance or palliative therapy. In total, 13 different DDR-related drugs have been investigated. Among these drugs, olaparib, ceralasertib, niraparib, talazoparib, veliparib, and stenoparib act as inhibitors targeting PARP, a key DDR-related protein. Additionally, ceralasertib, gartisertib, berzosertib, elimusertib, and tuvusertib act as ATR inhibitors; DAT-2645 targets PARG, adavosertib inhibits Wee1; and prexasertib functions as CHK1 inhibitor. These clinical trials have explored treatment regimens involving DDR-targeted drugs either as monotherapies or in combination with immunotherapy or chemotherapy. Identifying reliable biomarkers for DDR pathways may enhance patient stratification and optimize therapeutic outcomes.

### 4.4 Future investigation clues

Compared to previous studies on DNA repair deficiency in breast cancer, our analysis underscores a more focused exploration of this research field by examining publication trends, countries, authors’ impact, co-occurrence networks, article numbers from different institutions and co-occurrence networks, keywords, and references. This comprehensive approach reveals a broader and more influential research landscape. Additionally, several investigations have highlighted the role of the tumor microenvironment in modulating the response to DNA damage. The impact of environmental factors on DNA repair progress—such as ionizing radiation, chemical exposure, and the role of gut microbiota—has also been investigated to understand how these factors interact with genetic predispositions to influence breast cancer progression (53, 54). Emerging areas, such as the use of ctDNA to monitor specific DNA repair deficiencies, as well as advancements in monitoring techniques, warrant further exploration (55). Future investigations into DDR mechanisms—particularly pathways like NER and BER—in the context of ctDNA monitoring could provide valuable insights. Moreover, immunotherapy strategies may become increasingly relevant when considering the impact of environmental factors on DNA repair processes. Integrating genomic, transcriptomic, proteomic, and metabolomic data could offer a more comprehensive understanding of DDR mechanisms in breast cancer, potentially unveiling novel therapeutic targets and informing the development of personalized treatment approaches.

### 4.5 Limitations

Nevertheless, while bibliometric studies offer valuable insights, they also present limitations, such as bias toward publication frequency and the uneven impact of different research areas. To enhance the robustness of future analyses, it is essential to complement bibliometric data with qualitative assessments, including focus groups and expert interviews. Furthermore, as investigations continue to evolve, continuous efforts are needed to update bibliometric databases and analyses to capture emerging trends and breakthroughs in DDR-related breast cancer research. By synthesizing quantitative data with qualitative insights, stakeholders can adopt a more informed and strategic approach to advancing breast cancer studies, ultimately improving patient outcomes.

## 5 Conclusion

Research on DNA damage repair mechanisms in breast cancer has expanded significantly, providing deep insights into the etiology, progression, and treatment response of the disease. The interplay between genetic and environmental factors within DDR pathways highlights the complex nature of breast cancer. Increasingly, investigations—particularly those focusing on genetic predispositions, treatment strategies, and targeted therapies—hold great promise for improving patient outcomes and advancing personalized care. As we continue to advance our understanding of these mechanisms through this bibliometric study, our goal remains to translate scientific discoveries into tangible clinical benefits for patients.

## Data Availability

The raw data supporting the conclusions of this article will be made available by the authors, without undue reservation.
