# Correction: Knowledge mapping of global trends in DNA damage repair-related breast cancer research: a bibliometric study

**DOI:** 10.3389/fonc.2025.1646064

**Published:** 2025-09-09

**Authors:** Yajing Huang, Shumei Wei, Kaimin Hu, Xueping Xiang

**Affiliations:** ^1^ Department of Pathology, the Second Affiliated Hospital, Zhejiang University, School of Medicine, Hangzhou, China; ^2^ Department of Breast Surgery and Oncology, the Second Affiliated Hospital, Zhejiang University, School of Medicine, Hangzhou, China

**Keywords:** DNA damage repair, breast cancer, global trends, bibliometric study, CiteSpace, VOSviewer

There was a mistake in **Figure 9D** as published. In the published form of **Figure 9D**, two drugs somehow were not presented, including Stenoparib (distributed in one clinical trial) and Tuvusertib (distributed in one clinical trial). The corrected presentation of **Figure 9D** appears below.

**Figure 9:**
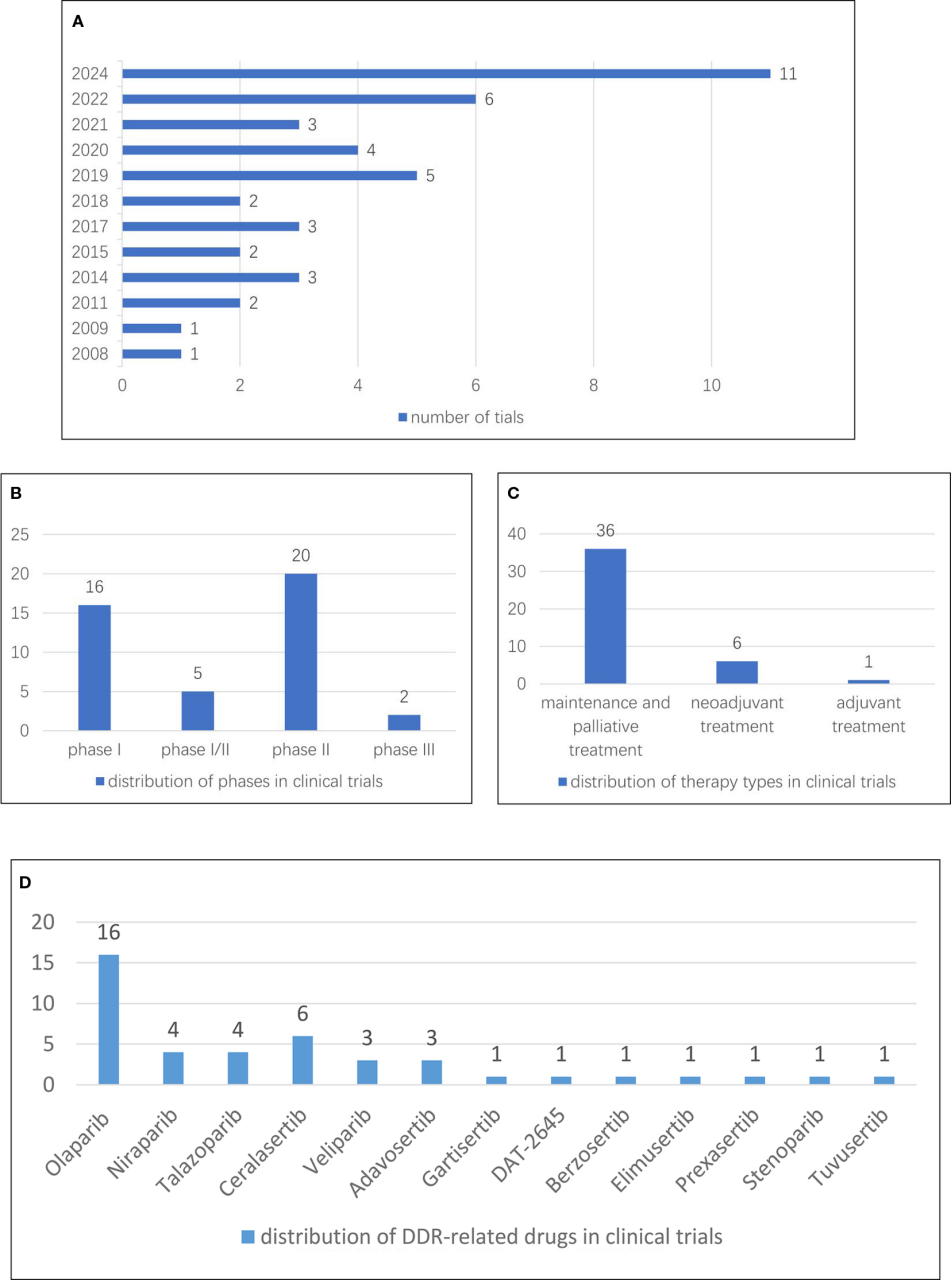
**(A)** Annual distribution of a number of clinical trials. **(B)** Distribution of phases in clinical trials. **(C)** Distribution of therapy types in clinical trials. **(D)** Distribution of DDR-related drugs in clinical trials.

The original version of this article has been updated.

